# The effect of thyme herb in diets for fattening pigs on their growth performance and health

**DOI:** 10.1371/journal.pone.0291054

**Published:** 2023-10-05

**Authors:** Anna Czech, Kamila Klimiuk, Iwona Sembratowicz

**Affiliations:** Department of Biochemistry and Toxicology, University of Life Sciences in Lublin, Lublin, Poland; University of Illinois, UNITED STATES

## Abstract

To enrich pork with valuable n-3 PUFA, it is common practice to include flaxseeds in the swine diet. However, due to the high susceptibility of these acids to oxidation, this treatment requires an additional supply of antioxidants. Thyme herb can be used for this purpose, which in addition to high antioxidant activity is characterized by numerous health-promoting properties. The present study aimed at evaluating the impact of the inclusion of 1% and 3% of thyme herb in mixtures with 4% of extruded flaxseeds as a source of n-3 PUFA on the performance results and health status of fatteners. The experiment was carried out on 120 weaners with an initial body weight of about 30 kg and kept until the end of fattening. They were divided into three experimental groups of 40 animals each (5 pens with 8 pigs in each). The control group (C) consisted of pigs receiving a base mixture with 4% of extruded flaxseeds. In the experimental groups, an additional 1% (T1) or 3% (T3) of thyme herb was added to the mixture. By supplementing the diet with 3% of thyme, an increase in average daily weight gain (P = 0.001) and a better feed conversion ratio (P < 0.001) were obtained. This could be the result of an improvement in the small intestine histology (greater villus height–P < 0.001) and better digestibility of basic nutrients (especially crude protein–P < 0.05) found in experimental animals. In addition to these effects, thyme herb supplementation contributed to the stimulation of immune mechanisms (increase in the number of WBC–P ≤ 0.05; plasma IgA ‐ P < 0.05 and IgG–P < 0.005, and the level of lysozyme–P < 0.05). The obtained results indicate the advisability of the use of thyme as a feed additive beneficially influencing the health and performance results in pigs. The obtained results indicate the advisability of the use of thyme as a feed additive beneficially influencing the health and performance results in pigs.

## Introduction

It is extremely important to include adequate levels of n-3 polyunsaturated fatty acids (PUFAs) in a well-balanced diet for both younger and older pigs. For this reason there is a clear trend of adding various significant sources of these fatty acids to feed. Rich sources of n-3 PUFAs include vegetable oils, e.g. hemp or rapeseed oil, animal oils, such as fish oil, and flaxseed or flaxseed oil [1].

Our previous research has shown that the inclusion of extruded flaxseed as a source of n-3 PUFAs in the diet of pigs improves the digestibility of basic nutrients (EE and NfD) and performance parameters, as well as meat quality (an increased concentration of n-3 in the tissues) and animal health [2]. However, our observations indicate that increased amounts of unsaturated fatty acids susceptible to oxidative changes, especially n-3 acids, necessitate additional intake of antioxidants. Natural antioxidants are recommended, especially herbs, which apart from antioxidant properties also exert numerous health-promoting effects. Herbs with antioxidant properties include oregano, turmeric, caraway, basil, ginger, rosemary, thyme, marjoram, and garlic [3]. The use of some of these in animal diets has been the subject of numerous studies, e.g. garlic, oregano and rosemary [4].

There are few reports, however, on the use of thyme herb in the diet of pigs. Thyme (*Thymus vulgaris*) is an aromatic plant of the family *Labiatae*. According to Dauqan and Abdullah [5], fresh thyme herb is characterized by one of the highest concentrations of antioxidant substances among herbs. These properties are mainly due to the presence of phenolic monoterpenes (thymol and carvacrol), which is part of the essential oil fraction. In addition, thyme contains other phenolics like rosmarinic acid, flavonoids (lutein, zeaxanthin, apigenin, luteolin, naringenin, and thymonin) and phenolic acids [6]. Apart from antioxidant activity, thyme exhibits antimicrobial and anti-inflammatory properties, and for this reason is used in the treatment of upper respiratory and gastrointestinal inflammation, as well as to induce excretion of intestinal parasitic worms [5]. Owing to the presence of so many bioactive substances, thyme is also counted among immunomodulatory plants, which translates to improved immunity in animals [7]. Another argument in favour of its use in animal diets is that it supports gastrointestinal function, e.g. by increasing secretion of digestive enzymes or exerting a spasmolytic effect [8]. Many studies in poultry have shown benefits in the form of improved rearing parameters as a result of the use of thyme herb or thyme oil in the diet [9]. The available literature lacks studies on the use of thyme herb in the diet of pigs.

Taking into account the anti-inflammatory and antioxidant properties of thyme, we assume that its inclusion in the feed mixture enriched with PUFA (extruded flaxseed) will have a positive effect on the intestinal histology, which will result in an increase in the digestibility of nutrients and, as a consequence, an improvement in the performance parameters of fattening pigs.

Therefore the aim of the study was to assess the effect of the inclusion of 1% or 3% thyme herb in diets with 4% extruded flaxseed as a source of n-3 PUFAs on the growth performance and health of fattening pigs.

## Material and methods

### Experimental design

The experiment was conducted on 120 piglets (Polish Large White x Neckar) weighing approximately 30 kg (about 84 days of age), which were kept until the end of the fattening period (about 110 kg BW; 168 days of age). The animals were divided into three experimental groups matched for sex and body weight. Each group consisted of 40 animals, were housed in 5 pens with 8 pigs in each (4 gilts and 4 barrows). In order to minimize the influence of factors that could affect the obtained result, variation in body weight within and between pen replicates and groups was minimized to the extent practically possible. The pigs were kept in pens on straw.

All methods were carried out in accordance with relevant guidelines and regulations regarding to life animal studies and the procedures complied with the Directive 2007/526/EC of the European Parliament and of the ouncil on the protection of animals used for scientifc purposes. All tests on animals were performed with the approval of the Local Ethics Committee on Animal Experimentation of the University of Life Sciences in Lublin, Poland (approval no. No. 64/2011).

### Animals and diet

In the presented experiment, the control group (group C) comprised animals receiving a diet with 4% extruded flaxseed but without thyme herb. In groups T1 and T3, 1% or 3% of thyme was added to the basal diet. The animals were fed complete Grower and Finisher diets *ad libitum*. The ingredient composition of the diets is presented in Table 1. The content of nutrients, amino acids, phosphorus and calcium in all groups met NCR nutritional requirements for pigs [10]. Thyme herb was introduced in place of barley in the feed mixtures for animals from groups T1 and T3. The content of bioactive substances and antioxidant indicators of thyme herb was presented in the work by Klimiuk et al. [11], and their levels were as follows: total polyphenols ‐ 12.32 mg GAE/g; total flavonoids ‐ 1.50 mg CE/g; ABTS^•+^ (2,2′-azinobis-(3-ethylbenzthiazolin-6-sulfonic acid) radical) - 2.11mmol Trolox/g; DPPH^•^ (2,2-diphenyl-1-picrylhydrazyl radical) - 1.76mmol Trolox/g.

**Table 1:** Composition and nutritive value of grower and finisher pig diets [11].

Ingredient	Thyme herb	Feeding groups^1^					
		Grower (25–70 kg BW)			Finisher (71–115 kg BW)		
		C	T1	T3	C	T1	T3
Ingredient (g/kg)							
Wheat		230	230	230	100	100	100
Rapeseed meal		80	80	80	120	122	125
Soybean meal		100	101	103	0	0	0
Thyme herb		0	10	30	0	10	30
Barley		483	472	450	647	635	612
Extruded flaxseed		40	40	40	40	40	40
Extruded soybean		20	20	20	20	20	20
Soya oil		20	20	20	0	0	0
Wheat bran		0	0	0	50	50	50
Dicalcium phosphate		3	3	3	0	0	0
Limestone		3	3	3	6	6	6
L-lysine chloride		1	1	1	2	2	2
Mineral-vitamin premix^2^		20	20	20	15	15	15
Total		1000	1000	1000	1000	1000	1000
Nutrient level (g/kg)							
Dry matter	994.3 (± 5.4)	893.5	893.6	893.6	893.8	893.7	893.9
Crude protein	58.7 (± 1.43)	169.4	169.3	169.2	150.2	150.1	150.1
Ether extract	39.2 (± 0.76)	56.1	56.1	56.1	38.2	38.1	38.3
Crude fibre	212.2 (± 16.50)	49.3	49.4	49.6	57.4	57.5	57.7
Total lysine		10.3	10.3	10.3	9.06	9.06	9.06
Methionine + cysteine		6.52	6.52	6.52	5.89	5.89	5.89
Total phosphorus		5.57	5.57	5.58	5.17	5.17	5.17
Calcium		7.12	7.12	7.12	6.49	6.49	6.49
ME, MJ		13.07	13.07	13.06	12.56	12.56	12.55

^1^There were 3 dietary treatments: a control diet (C), and diets T1 and T3 with 1% or 3% thyme, respectively.

^2^1 kg of mineral-vitamin premix contained: vitamin A 600 000 IU, D_3_ 60 000 IU, E 3000 mg, K_3_ 120 mg, B_1_ 120 mg, B_2_ 240 mg, B_6_ 240 mg, nicotinic acid 1600 mg, pantothenic acid 800 mg, folic acid 160 mg, biotin 10 mg, B_12_ 1.6 mg, choline chloride 12 g, Mg 0.8 g, Fe 6 g, Zn 5.6 g, Mn 2.4 g, Cu 6.4 g, J 40 mg, Se 16 mg, Co 16 mg.

The choice of thyme herb was influenced by a pilot experiment in which the dietary preferences of piglets were observed. In that experiment piglets received diets with a 3% share of herbs with antioxidant properties: thyme, caraway, coriander, or purple coneflower. The diet most preferred by the piglets was observed to be the one with thyme herb.

The thyme herb used in the experiment was obtained from a single farm on which thyme was cultivated. Dried thyme herb was added to the diets when they were being prepared in the feed mixer.

### Growth performance parameters

All animals were weighed prior to the start of the experiment, and again when the diet was changed from grower to finisher (at about 70 kg BW) and at the end of the experiment (about 110 kg BW). Then average daily weight gain (ADG) was calculated. Feed intake (FI) was recorded for each pen throughout the experiment. The FI values were calculated per pig (by dividing by the number of pigs per pen). The FI and feed conversion ratio (FCR) are expressed as the pen average for each period. At the end of the experiment (about 110 kg BW) the animals were slaughtered with the use of the electric shock method.

### Analytical methods

Before the start of each stage of the experiment, the thyme herb and the experimental diets were sampled–from three different places in the batch of feed and from three different places in the batch of thyme herb–for analysis of proximate chemical composition, i.e. the content of dry matter (103˚C for 20h), crude protein (procedure 950.36; (N×6.25) determined by the Kjeldahl method), ether extract (procedure 935.38), and crude fibre (procedure 962.09), according to AOAC [12]. The diets were also analysed for the content of total phosphorus (procedure 964.06) and calcium (procedure 927.02) according to AOAC methods [12].

Contents of amino acids were determined on a Sykam Amino Acid Analyzer (Laserchrom HPLC Laboratories Ltd. Inc., Rochester, UK). Prior to analysis, the samples were hydrolysed with 6 N HCl at 110˚C for 24 h. Methionine and cysteine were analysed as methionine sulfone and cysteic acid after cold performic acid oxidation overnight prior to hydrolysis.

Prior to analysis, the samples were homogenized using a BUCHI mixer B-400 with ceramic blades. All analyses were performed in triplicate.

#### Digestibility analysis

At the end of the growing period (grower: 65–75 kg BW) and fattening period (finisher: 100–110 kg BW), 3 g of chromium oxide (Cr_2_O_3_) per kg of diet was added to the feed of all groups as a digestibility marker, prior to pelleting [13]. During both periods 6 barrows from each group underwent 6-day adaptation, after which excreted faeces from each pig were collected and weighed for 4 days, at the same time each day. About 20% of the faeces collected from each pig was removed and placed in plastic bags. The samples were stored at a temperature of +4ºC until they were transported to the laboratory. The excrement collected from each pig over 4 days was thoroughly mixed, and samples were taken. The averaged samples prepared in this manner from the 4-day collection were used for chemical analyses. Dried and homogenized faeces samples were analysed for content of basic chemical components, i.e. dry matter, crude protein (TP), ether extract (EE), and crude fibre (Cfibre), according to AOAC [12] methods. The content of nitrogen-free extract (NfE) was calculated as well.

At the end of the digestibility studies, 2 barrows in each group were left in the pen for one day and faeces were collected 24 hours a day (individually). It was used to determine the Cr_2_O_3_ index needed to determine the recovery rate of the digestibility marker.

The content of chromium oxide in the diets and faeces samples was determined according to Suzuki and Early [14].

The apparent total digestibility (ATTD) of the nutrients were calculated according to the following formula:

$$\text{ATTD}\;=\;100\;\left(1-\left(\text{Ni}\;\text{Md}\right)\;\text{/}\;\left(\text{Nd}\;\text{Mi}\right)\right)$$

where Nd is the concentration of a given nutrient in the diet, Ni is the concentration of the nutrient in the faeces, Md is the concentration of the marker (chromium oxide) in the diet, and Mi is the concentration of the marker in the faeces (all values expressed in g/kg DW) [13].

#### Blood analysis

Blood was collected twice from the same pigs that were used for digestibility analysis (6 pigs from each group), at a body weight of about 70 and 100 kg. The animals had no access to feed for 12 h before blood sampling. Blood was collected by a veterinarian from the jugular vein into 10 ml heparinized tubes.

The blood samples were centrifuged at 3500 g for 15 min to obtain plasma, which was frozen (–20°C) until analysis.

White blood cell parameters, i.e. white blood cell count (WBC) and differential leukocyte count–the percentages of granulocytes (GRA), lymphocytes (LYM) and MID (sum of eosinophils + basophils + monocytes), were analysed in whole blood using an ABACUS-Vet analyser.

The titre of IgG (QY-E40124), IgA (QY-E40199), IgM (QY-E40123) immunoglobulins and the content of lysozyme (QY-E40200) and IL-6 (QY-E40023) in the plasma were determined by an immunoenzymatic ELISA assay using kits from AssayGenie (Dublin, Ireland)

#### Post-mortem analysis

Carcass analysis was performed after slaughter (∼110 kg) on 6 pigs from each group–the same animals used for the blood and digestibility analyses. Carcass analysis involved determination of dressing percentage (%); meat percentage in the ham (%); loin area (cm^2^); lean meat content; average backfat thickness from 5 measurement points (BT 5); and the weight of the kidney, liver and heart. The intestines were also collected for morphometric analysis.

#### Morphometric analysis of the intestines

Intestinal samples for morphometric analysis were fixed in 10% buffered formalin, processed in a tissue processor (TP 1020, Leica Biosystems, Nussloch, Germany), and embedded in paraffin. Sections 3 μm thick were prepared from the paraffin blocks and stained by standard H&E (haematoxylin and eosin) staining. Morphometric analysis of the slides was then performed using the LUCIA NIS Elements BR 2.20 image processing and analysis system (Nikon Corporation, Tokyo, Japan), according to methods described by Hedemann et al. [15]. Measurements were made under 40x magnification in 6 randomly chosen fields of view. The length of the villi and their width at the base were measured in the ileum, as well as the depth and width of the crypts and the width of the muscular layer. Analogous measurements were made of the crypts and muscular layer in the caecum and colon.

### Statistical analysis of results

All data are expressed as means and SEM (standard error of the mean).

Data were analysed by ANOVA. The performance parameters were measured based on replication groups (animals were kept in 5 pens; 8 animal/1 pen). For slaughter parameters single animal (randomly chosen from each replication group) was treated as a scientific unit, it allowed to have 6 values of each parameters per group. It is minimal number of samples for statistical purposes and also according to the Local Ethical Committee, approval. Resolution No. 64/2011.

To determine the effect of the addition of thyme and the level of thyme, the data were analyzed using pre-planned contrasts according to the following model:

T = difference between control group and supplementation of thyme (C vs T1+T3)

D = difference between treatment with supplementation level of thyme (T1 vs T3).

Treatment means were compared using Tukey’s multiple comparison test in Statistica 13 (Dell Software Inc., USA).

## Results

The analysis showed that the thyme herb used in the experiment contained 58.7 g/kg crude protein, 39.2 g/kg ether extract, and 212.2 g/kg crude fibre (Table 1).

### Fattening pigs production indices, nutrient digestibility and dressing analysis

The inclusion of 3% thyme in diets for fatteners significantly increased their body weight in both the grower and finisher period (*P* = 0.005 and *P* = 0.001, respectively), as well as their average daily gains (ADG), (*P* = 0.01 and *P* = 0.03, respectively), compared to the control group and group T1 (Table 2). FI in group T3 was significantly higher than in group C during the grower period (*P* = 0.038), and compared to groups C and T1 during the finisher period (*P* = 0.014). Animals from the group receiving 3% thyme had a significantly lower feed conversion ratio (FCR) than in the control group and group T1 in the finisher period (*P* < 0.001). The use of thyme significantly influenced mean ADG (*P* = 0.007) and FCR (*P* = 0.003) in comparison with the control group (C *vs*. T), (Table 2). The dose of thyme significantly influenced average BW (grower *P* = 0.009; finisher *P* = 0.014), mean ADG (*P* = 0.026) and mean FCR (*P* < 0.001).

**Table 2:** Performance parameters of fattening pigs.

Item^1^	Feeding groups^2^			SEM^3^	*P* value		
	C	T1	T3		TRT^4^	T^5^	D^6^
BW (kg)							
Start	30.36	30.07	30.56	0.110	0.196	0.762	0.113
Grower	75.71^b^	76.45^b^	79.62^a^	0.531	0.005	0.051	0.009
Finisher	108.42^b^	110.86^b^	115.99^a^	0.895	0.001	0.009	0.014
ADG (kg per day)							
Grower	0.889^b^	0.909^ab^	0.962^a^	0.01	0.010	0.038	0.027
Finisher	0.839^b^	0.882^ab^	0.932^a^	0.019	0.030	0.019	0.229
Mean	0.867^b^	0.898^b^	0.949^a^	0.01	0.001	0.007	0.026
FI (kg)							
Grower	2.30^b^	2.38^ab^	2.51^a^	0.034	0.038	0.049	0.067
Finisher	2.91^a^	2.86^a^	2.44^b^	0.073	0.014	0.111	0.017
Mean	2.53	2.55	2.48	0.027	0.547	0.769	0.327
FCR (kg/kg)							
Grower	2.6	2.61	2.61	0.011	0.955	0.769	0.915
Finisher	3.49^a^	3.25^a^	2.62^b^	0.083	<0.001	0.001	<0.001
Mean	2.92^a^	2.84^a^	2.61^b^	0.031	<0.001	0.003	<0.001

^1^FI = feed intake; ADG = average daily gain; FCR = feed conversion ratio

^a–b^ Means with the same superscript are statistically the same across all 3 treatments (*P* > 0.05) based on Tukey’s *post hoc* test.

^2^There were 3 dietary treatments: a control diet (C), and diets T1 and T3 with 1% or 3% thyme, respectively.

^3^SEM–standard error of the means

^4^TRT–*P* value for overall effect of dietary treatment (diets 1–3)

^5^T –*P* value for effect of thyme *vs*. control (diet 1 *vs*. 2 and 3)

^6^D –*P* value for effect of level of thyme (1% *vs*. 3%)

Faecal digestibility of crude protein was significantly higher in pigs receiving a diet with 3% thyme than in groups C and T1 during the entire experiment (*P* = 0.030 grower and *P* = 0.027 finisher), (Table 3). The inclusion of 3% thyme in diets for pigs during the grower period significantly increased faecal digestibility of EE compared to group T1 (*P* = 0.050) and of Cfibre compared to group C (*P* = 0.023). The dose of thyme significantly influenced CP (finisher *P* = 0.023), EE (grower *P* = 0.046; finisher *P* = 0.017).

**Table 3:** Apparent total digestibility of nutrients (ATTD) in the diet of fatteners and mean recovery of markers in faeces (%).

Item^1^	Feeding groups^2^			SEM^3^	*P* value		
	C	T1	T3		TRT^4^	T^5^	D^6^
Faecal recovery	96.20	96.22	96.19	0.335	0.564	0.489	0.745
CP							
Grower	72.17^b^	72.70^b^	73.91^a^	0.296	0.030	0.069	0.108
Finisher	72.62^b^	72.52^b^	74.15^a^	0.413	0.027	0.442	0.032
EE							
Grower	64.12^ab^	63.95^b^	65.45^a^	0.292	0.050	0.379	0.046
Finisher	65.45	64.78	66.63	0.36	0.093	0.76	0.017
Cfibre							
Grower	24.40^b^	25.52^ab^	25.92^a^	0.319	0.023	0.044	0.637
Finisher	25.43	25.15	25.20	0.222	0.887	0.62	0.942
NfE							
Grower	89.03	90.98	90.18	0.289	0.095	0.084	0.157
Finisher	91.35	91.15	91.80	0.190	0.394	0.772	0.115

^1^CP- crude protein, EE ‐ ether extract, Cfibre ‐ crude fibre, NfE ‐ nitrogen-free extract

^a–b^ Means with the same superscript are statistically the same across all 3 treatments (*P* > 0.05) based on Tukey’s *post hoc* test.

^2^There were 3 dietary treatments: a control diet (C), and diets T1 and T3 with 1% or 3% thyme, respectively.

^3^SEM–standard error of the means

^4^TRT–*P* value for overall effect of dietary treatment (diets 1–3).

^5^T –*P* value for effect of thyme *vs*. control (C *vs* T1+T3)

^6^D –*P* value for effect of level of thyme (T1 *vs* T3)

The inclusion of thyme in diets for fatteners did not significantly influence carcass analysis parameters, i.e. dressing percentage, meat % in ham, lean meat content, BT5, kidney weight, liver weight, and heart weight (Table 4). It only significantly increased the loin area compared to group C (*P* = 0.042).

**Table 4:** Dressing percentage and organ weights of fatteners (n = 6 each group).

Item^1^	Feeding groups^2^			SEM^3^	*P* value		
	C	T1	T3		TRT^4^	T^5^	D^6^
Dressing percentage, %	78.81	79.47	79.10	0.149	0.201	0.143	0.299
Meat % in ham	79.06	78.85	78.95	0.079	0.576	0.342	0.649
Loin area, cm^2^	58.90^b^	61.81^ab^	64.60^a^	1.03	0.042	0.046	0.239
Lean meat content, %	61.55	62.16	61.97	0.264	0.665	0.378	0.736
BT5, cm	2.53	2.47	2.23	0.063	0.125	0.194	0.131
Kidney weight, g	2.65	1.88	1.74	0.270	0.364	0.148	0.871
Liver weight, kg	1.29	1.10	1.01	0.144	0.750	0.464	0.837
Heart weight, g	315.2	322.9	315.8	1.12	0.143	0.766	0.135

^1^BT ‐ average backfat thickness from 5 measurement points

^a–b^ Means with the same superscript are statistically the same across all 3 treatments (*P* > 0.05) based on Tukey’s *post hoc* test.

^2^There were 3 dietary treatments: a control diet (C), and diets T1 and T3 with 1% or 3% thyme, respectively.

^3^SEM–standard error of the means

^4^TRT–*P* value for overall effect of dietary treatment (diets 1–3).

^5^T –*P* value for effect of thyme *vs*. control (C *vs* T1+T3)

^6^D –*P* value for effect of level of thyme (T1 *vs* T3)

### Histological analysis of the intestines

Histological analysis of the sections of the pigs’ intestines showed that the inclusion of 3% thyme significantly increased the length and width of the villi and the thickness of the muscularis externa in the ileum relative to the control group (P ≤ 0.001, P = 0.049 and P = 0.021, respectively), (Table 5). The use of 3% thyme (T3) significantly increased the depth and width of the crypts in the ileum (P = 0.039 and P = 0.040, respectively). The villus VH/CD depth ratio and muscularis externa in the ileum of the pigs in the groups receiving thyme (T) was significantly higher than in the control group (P < 0.001 and P = 0.040, respectively). In the caecum and colon, the inclusion of thyme in the diet significantly increased the depth of the crypts compared to the control group (P = 0.035 and P = 0.011, respectively), (Table 5).

**Table 5:** Histological analysis of sections of the intestines of pigs (n = 6 each group).

Item^1^		Feeding groups^2^			SEM^3^	*P* value		
		C	T1	T3		TRT^4^	T^4^	D^5^
Ileum (μm)								
Villus	height	341.2^c^	451.8^b^	505.2^a^	6.33	<0.001	<0.001	0.013
	width	143.8^b^	150.0^ab^	178.7^a^	4.70	0.049	0.741	0.036
Crypt	depth	337.0^b^	330.0^b^	364.8^a^	3.69	0.039	0.028	0.867
	width	53.96^b^	50.86^b^	67.09^a^	2.96	0.040	0.049	0.612
VH/CD		1.01^b^	1.37^a^	1.38^a^	0.043	0.010	<0.001	0.243
Muscularis externa		363.9^b^	405.8^a^	435.8^a^	8.95	0.021	0.040	0.082
Caecum (μm)								
Crypt	depth	420.6^b^	452.4^ab^	497.5^a^	5.01	0.035	0.027	0.546
	width	72.70	74.58	73.53	3.26	0.384	0.293	0.226
Muscularis externa		207.30	230.2	220.7	15.20	0.853	0.599	0.835
Colon (μm)								
Crypt	depth	476.9^b^	478.6^b^	561.5^a^	9.ll02	0.011	0.048	0.088
	width	54.43	59.00	63.29	2.58	0.414	0.248	0.525
Muscularis externa		178.3	238.1	190.3	15.28	0.258	0.289	0.226

^1^VH ‐ villus height, CD ‐ crypt depth

^a–b^ Means with the same superscript are statistically the same across all 3 treatments (*P* > 0.05) based on Tukey’s *post hoc* test.

^2^There were 3 dietary treatments: a control diet (C), and diets T1 and T3 with 1% or 3% thyme, respectively.

^3^SEM–standard error of the means

^4^TRT–*P* value for overall effect of dietary treatment (diets 1–3).

^5^T –*P* value for effect of thyme *vs*. control (C *vs* T1+T3)

^6^D –*P* value for effect of level of thyme (T1 *vs* T3)

### Blood indices

In each stage of the experiment, the blood of pigs receiving a diet with 3% thyme had significantly higher WBC and LYM counts compared to the control group (*P* ≤ 0.05), (Table 6). The lymphocyte count was also significantly higher in the blood of pigs from group T3 compared to group T1 (*P* = 0.02 –grower and *P* = 0.048 finisher). Grower pigs from group T3 also had a significantly lower number of granulocytes than pigs from groups C and T1 (*P* = 0.048).

**Table 6:** Haematological parameters of the blood of fattening pigs (n = 6 each group).

Item^1^	Feeding groups^2^			SEM^3^	*P* value		
	C	T1	T3		TRT^4^	T^5^	D^6^
WBC (10^9^/L)							
Grower	16.41^b^	17.44^ab^	18.35^a^	0.226	0.050	0.02	0.321
Finisher	17.28^b^	18.84^ab^	19.77^a^	0.166	0.042	0.037	0.330
GRA (%)							
Grower	52.12^a^	51.75^a^	45.35^b^	0.897	0.048	0.224	0.028
Finisher	52.94	53.99	48.18	1.28	0.142	0.224	0.106
MID (%)							
Grower	2.78	3.48	2.01	0.316	0.166	0.953	0.049
Finisher	1.82	1.79	1.77	0.164	0.991	0.953	0.953
LYM (%)							
Grower	45.09^b^	44.78^b^	52.63^a^	1.00	0.049	0.278	0.020
Finisher	45.24^b^	44.23^b^	50.05^a^	0.713	0.043	0.278	0.048

^1^WBC–while blood cells; GRA–granulocytes; LYM–lymphocytes; MID–sum of monocytes + eosinophils + basophils

^a–b^ Means with the same superscript are statistically the same across all 3 treatments (*P* > 0.05) based on Tukey’s *post hoc* test.

^2^There were 3 dietary treatments: a control diet (C), and diets T1 and T3 with 1% or 3% thyme, respectively.

^3^SEM–standard error of the means

^4^TRT–*P* value for overall effect of dietary treatment (diets 1–3).

^5^T –*P* value for effect of thyme *vs*. control (C *vs* T1+T3)

^6^D –*P* value for effect of level of thyme (T1 *vs* T3)

The blood plasma of grower and finisher pigs receiving a diet with 3% thyme had significantly higher levels of IgG, IgA and lysozyme in comparison with animals from the control group (*P* < 0.05), (Table 7).

**Table 7:** Immune parameters of the blood of fattening pigs (n = 6 each group).

Item^1^	Feeding groups^2^			SEM^3^	*P* value		
	C	T1	T3		TRT^4^	T^5^	D^6^
IgG (mg/mL)							
Grower	18.25^b^	19.69^b^	23.40^a^	0.150	0.005	0.012	0.019
Finisher	13.18^c^	16.70^b^	19.31^a^	0.716	<0.001	0.031	0.015
IgA (μg/mL)							
Grower	550.1^b^	599.7^ab^	654.4^a^	0.741	0.031	0.031	0.203
Finisher	563.2^b^	597.6^ab^	628.6^a^	9.55	0.010	0.025	0.143
IgM (μg/mL)							
Grower	270.6	286.7	311.2	1.13	0.128	0.282	0.366
Finisher	299.7	303.9	320.9	1.93	0.099	0.108	0.083
Lysozyme (mg/mL)							
Grower	1.99^b^	1.87^b^	2.71^a^	0.282	0.011	0.108	0.016
Finisher	1.75^b^	2.00^b^	3.05^a^	0.155	<0.001	0.282	<0.001
IL6 (pg/mL)							
Grower	5.46	4.94	4.86	0.090	0.087	0.075	0.211
Finisher	4.74	4.74	4.96	0.108	0.654	0.112	0.322

^1^ IL6 ‐ Interleukin-6

^a–b^ Means with the same superscript are statistically the same across all 3 treatments (*P* > 0.05) based on Tukey’s *post hoc* test.

^2^There were 3 dietary treatments: a control diet (C), and diets T1 and T3 with 1% or 3% thyme, respectively.

^3^SEM–standard error of the means

^4^TRT–*P* value for overall effect of dietary treatment (diets 1–3).

^5^T –*P* value for effect of thyme *vs*. control (C *vs* T1+T3)

^6^D –*P* value for effect of level of thyme (T1 *vs* T3)

## Discussion

In the first part of the research, published by Klimiuk et al. [11] and Czech et al. [16], animals in experimental groups were given diets with 2%, 4%, or 6% extruded flaxseed instead of barley. It was demonstrated that the addition of 4% extruded flaxseed proved to be the most promising solution for addressing the deficiency of polyunsaturated fatty acids in diets for fattening pigs. However, due to the susceptibility of PUFA to oxidation, we decided to introduce thyme herb into the mixture, characterized by antioxidant properties.

There are numerous reports indicating that diets containing thyme herb or thyme oil improve the growth performance of animals raised for meat, mainly poultry [9]. However, the available literature describes few experiments using thyme herb in the diet of pigs. A study by Jugl-Chizzola et al. [17] in which 1% thyme was included in the diet of weaners confirmed its antibacterial effectiveness (*in vitro*) against haemolysing strains of *E*. *coli* isolated from them, but showed no effect on growth performance. Experiments on the effectiveness of the use of components of essential oils in diets for piglets weaned from the sow, such as thymol or carvacrol, do not yield clear conclusions. Some studies have shown no effect of these substances on growth performance [18], while others have shown beneficial effects. Studies by Li et al. [19] found that weaned piglets receiving essential oil blend containing thymol and cinnamaldehyde had significantly higher daily gains (ADG) than the unsupplemented group. These relationships were also noted in the present study, in which pigs receiving 3% thyme herb in the diet had significantly higher daily gains and a better feed conversion ratio than pigs in the control group, but also compared to the pigs receiving the smaller, 1.5% share of thyme herb. According to Hashemi and Davoodi [20], substances present in herbs possess flavoring properties that improve the taste and smell of feed, thereby increasing feed intake, which in turn translates to improved weight gain and feed conversion. According to Windisch et al. [21], used as supplements they can stimulate the production of digestive enzymes such as trypsin and amylase, thus improving the degree of utilization of nutrients. Research on chickens showed that administration of a mixture of thymol and carvacrol (1:1) results in an increase in the activity of trypsin as well as lipase and protease [22].

The improved growth performance in the group of pigs receiving a diet with 3% thyme herb was likely due to the improvement in the digestibility of crude protein as well as ether extract and Cfibre, which was particularly evident in the first stage of the fattening period. The study by Li et al. [19], cited above, showed that the use of essential oil containing thymol and cinnamaldehyde in diets for piglets increased the digestibility of dry matter and crude protein. Improved nutrient digestibility in fattening pigs receiving feed with a formulation containing extracts of thyme, rosemary, oregano and kaolin was reported by Yan et al. [23]. Amad et al. [24] found that diets for chickens including a multi-component phytogenic feed additive containing essential oils, e.g. from thyme and anise, significantly increases the apparent ileal digestibility of crude fat, crude protein, and also minerals. Almanea et al. [8] report that research on animal models has shown that flavonoids in thyme exert an effect that relaxes the smooth muscles of the ileum, acting as blockers of histamine and acetylcholine receptors and/or as calcium channel antagonists [25]. Owing to the spasmolytic properties of the active substances in thyme it has a beneficial effect on passage of digesta and ensures appropriate interactions between feed and digestive enzymes.

Thyme herb did not affect most of the parameters of the carcass analysis in the present study or in a study by Rhouma et al. [26] in rabbits receiving thymol. It should be noted, however, that 3% thyme herb in the diet of pigs caused a significant increase in the loin area, which is a very positive effect for the breeder and consumer. Similar results for this parameter were obtained by Korniewicz et al. [27] in fattening pigs receiving a diet supplemented with a multi-component herbal preparation (500 mg/kg) containing mainly thymol, cineol and carvacrol. A beneficial effect on dressing percentage and the yield of breast, leg and wing muscles was obtained by El-Ghousein and Beitawi [28] in an experiment in chickens receiving 1.5–2% dried crushed thyme in their feed.

Improved nutrient digestibility and fattening efficiency may also be linked to the beneficial effect of the active substances in thyme on the histomorphology of the intestine [8]. The morphometric parameters of the small intestine, such as villus height and width, crypt depth, and the ratio of villus height to crypt depth, were much more favourable in the pigs receiving a diet with 3% thyme. These parameters determine the amount of digestive enzymes secreted, the absorptive surface, and transport of nutrients. An experiment in chickens, in which thyme extract was used in combination with thyme powder, confirmed their beneficial effect on the histomorphology of the small intestine [29]. Similar positive effects on intestinal morphology have been obtained in research in which piglets received essential oil containing thymol and cinnamaldehyde [19].

Many studies indicate that the presence of polyphenolic compounds in thyme is responsible for improvement of the immune response [22]. The present study showed a significant increase in the number of lymphocytes in the blood of pigs receiving a diet with 3% thyme herb, corresponding with a significantly higher level of class G and A immunoglobulins, as well as an increase in the lysozyme level in the blood plasma, which indicates stimulation of immune processes in the pigs. A study by Abdel-Ghaney et al. [30] using chickens showed that powdered thyme added to the diet modified immune parameters (levels of IgG and IgM in the blood), and that the effects were dependent on the amount of the additive (0.5–1.5%) and the duration of supplementation (3 or 5 weeks). The same experiment showed that the presence of thyme significantly increased the concentrations of cytokines: IFN-gamma (after 5 weeks of supplementation) and IL-10 (1.5%). Khazdair et al. [7] performed an extensive review of research on the immunomodulatory and anti-inflammatory effects of some medicinal herbs. They concluded that thyme and its most important component, i.e. thymol, have the capacity to reduce the expression of pro-inflammatory cytokines IL-6, IL-1β and TNFα. A study using albino rats [31] showed that the use of thyme also increased the WBC count, which was confirmed in our experiment. However, thyme did not affect the level of pro-inflammatory IL-6 in the blood of pigs. Li et al. [19] reported that weaned piglets whose diet was supplemented with essential oil containing thymol with cinnamaldehyde showed a significantly increase in lymphocyte proliferation, but not in plasma levels of IgG, IgM and IgA.

## Conclusion

In this study the inclusion of 3% thyme herb in the diet enriched with PUFA (extruded flaxseed) of fattening pigs improved the histology of the intestinal over its entire length, causing a significant increase in villus length which translated to improved nutrient digestibility and production efficiency. It also stimulated immune processes, resulting in increased levels of class G and A immunoglobulins and lysozyme in the blood plasma.

Improved feed intake corresponding to better weight gains in pigs receiving diets with 3% thyme herb, as well as stimulation of immune processes, suggests that this is a promising phytobiotic that can be used throughout the fattening period.

## Supporting information

S1 File (XLS)
